# Effectiveness of a fourth SARS‐CoV‐2 vaccine dose in previously infected individuals from Austria

**DOI:** 10.1111/eci.14136

**Published:** 2023-11-30

**Authors:** Alena Chalupka, Lukas Richter, Ali Chakeri, Ziad El‐Khatib, Verena Theiler‐Schwetz, Christian Trummer, Robert Krause, Peter Willeit, Bernhard Benka, John P. A. Ioannidis, Stefan Pilz

**Affiliations:** ^1^ Institute for Surveillance & Infectious Disease Epidemiology, Austrian Agency for Health and Food Safety (AGES) Vienna Austria; ^2^ Institute of Statistics, Graz University of Technology Graz Austria; ^3^ Center for Public Health, Medical University Vienna Vienna Austria; ^4^ Department of Internal Medicine, Division of Endocrinology and Diabetology Medical University of Graz Graz Austria; ^5^ Department of Internal Medicine, Division of Infectious Diseases Medical University of Graz Graz Austria; ^6^ Institute of Health Economics, Medical University of Innsbruck Innsbruck Austria; ^7^ Department of Public Health and Primary Care University of Cambridge Cambridge UK; ^8^ Ignaz Semmelweis Institute, Interuniversity Institute for Infection Research Vienna Austria; ^9^ Departments of Medicine, Epidemiology and Population Health, Biomedical Data Science, and Statistics and Meta‐Research Innovation Center at Stanford (METRICS) Stanford University Stanford California USA

**Keywords:** booster, COVID‐19, mortality, national, SARS‐CoV‐2, vaccine

## Abstract

**Introduction:**

Evidence is limited on the effectiveness of a fourth vaccine dose against coronavirus disease 2019 (COVID‐19) in populations with prior severe acute respiratory syndrome coronavirus 2 (SARS‐CoV‐2) infections. We estimated the risk of COVID‐19 deaths and SARS‐CoV‐2 infections according to vaccination status in previously infected individuals in Austria.

**Methods:**

This is a nationwide retrospective observational study. We calculated age and gender adjusted Cox proportional hazard ratios (HRs) of COVID‐19 deaths (primary outcome) and SARS‐CoV‐2 infections (secondary outcome) from 1 November to 31 December 2022, primarily comparing individuals with four versus three vaccine doses. Relative vaccine effectiveness (rVE) was calculated as (1‐HR) X 100.

**Results:**

Among 3,986,312 previously infected individuals, 281,291 (7,1%) had four and 1,545,242 (38.8%) had three vaccinations at baseline. We recorded 69 COVID‐19 deaths and 89,056 SARS‐CoV‐2 infections. rVE for four versus three vaccine doses was −24% (95% CI: −120 to 30) against COVID‐19 deaths, and 17% (95% CI: 14–19) against SARS‐CoV‐2 infections. This latter effect rapidly diminished over time and infection risk with four vaccinations was higher compared to less vaccinated individuals during extended follow‐up until June 2023. Adjusted HR (95% CI) for all‐cause mortality for four versus three vaccinations was 0.79 (0.74–0.85).

**Discussion:**

In previously infected individuals, a fourth vaccination was not associated with COVID‐19 death risk, but with transiently reduced risk of SARS‐CoV‐2 infections and reversal of this effect in longer follow‐up. All‐cause mortality data suggest healthy vaccinee bias.

## Graphical abstract

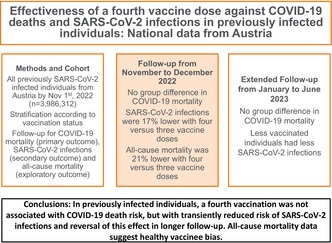

## 1 INTRODUCTION

In 2022, infection fatality rates due to severe acute respiratory syndrome coronavirus 2 (SARS‐CoV‐2) significantly declined suggesting coronavirus disease 2019 (COVID‐19) pandemic transitioning into endemicity.^1^, ^2^ By end 2022, the vast majority of the global population had already acquired some immune protection against SARS‐CoV‐2 by previous vaccinations and/or infections.^3^ Measures against SARS‐CoV‐2 including vaccine policy should be critically re‐assessed for their risk‐to‐benefit ratio.

One major issue is the efficacy of a fourth mRNA vaccine dose (second booster) against SARS‐CoV‐2, and whether and for whom such a vaccination is indicated.^2^, ^4^ Epidemiological studies suggest that four versus three vaccine doses significantly reduce SARS‐CoV‐2 infection rates, and prevent severe COVID‐19 and related deaths in populations largely free of previous SARS‐CoV‐2 infections, in particular in nursing home residents.^2^, ^5^, ^6^, ^7^, ^8^, ^9^, ^10^, ^11^, ^12^ Data on the effectiveness of the fourth SARS‐CoV‐2 vaccine dose in previously infected individuals are scarce and mainly restricted to old populations without specific data on COVID‐19 deaths and all‐cause mortality.^9^, ^10^, ^13^ In this context, it should be considered that natural immunity derived from previous infections, vaccine induced, and hybrid immunity derived from a previous infection and vaccination, may have a more sustainable protection against severe and lethal COVID‐19 than against SARS‐CoV‐2 infections per se.^14^, ^15^, ^16^, ^17^ As most SARS‐CoV‐2 infections are asymptomatic or mild in an endemic phase, effectiveness of vaccinations should be primarily evaluated according to hard clinical outcomes (e.g. COVID‐19 deaths), than just by SARS‐CoV‐2 positive tests.^2^, ^18^

In Austria, the country with the worldwide highest SARS‐CoV‐2 testing frequency per person, the national vaccine committee recommended a fourth vaccine dose for all individuals aged 12 years and older in August, 2022.^2^ Approximately 12% of the national population received this vaccination by the end of October, enabling evaluation of vaccination effectiveness. Here, we considered the entire general population of Austria with a previous SARS‐CoV‐2 infection. As primary outcomes, we calculated relative risks of COVID‐19 deaths in groups according to vaccination status, focusing primarily on four versus three doses. Respective relative risks for SARS‐CoV‐2 infections and all‐cause deaths were calculated as secondary and exploratory outcomes, respectively.

## 2 METHODS

### 2.1 Study design, procedures and participants

In this retrospective population‐based observational study, we used national health data from the Austrian epidemiological reporting system (*Epidemiologisches Meldesystem*; EMS) provided by the Austrian Agency for Health and Food Safety (*Österreichische Agentur für Gesundheit und Ernährungssicherheit*; AGES), that records data of all individuals with a documented SARS‐CoV‐2 infection.^19^ Unique personal identifiers were used to match the EMS data with individual all‐cause mortality data provided by Statistics Austria (covering until end‐2022) and with individual vaccination data provided by the national COVID‐19 vaccine registry.^19^ Residency in Austria was verified by checking the Central Registry of Residence in Austria at the date of SARS‐CoV‐2 infection and nursing home residency was checked by postal address at time of infection. No sample size calculation was performed prior to study initiation. We followed the strengthening the reporting of observational studies in epidemiology (STROBE) checklist (Table S1). Ethical approval was obtained from the ethics committee at the Medical University of Graz (no. 33–144 ex 20/21). The planning conduct and reporting of this study was in line with the Declaration of Helsinki, as revised in 2013.

The study population encompasses all previously SARS‐CoV‐2 infected residents (regardless of symptoms) in Austria, who had an entry of their infection into the EMS, and who were alive on 1 November 2022. According to the widely used definition that SARS‐CoV‐2 re‐infections require two positive tests separated by more than 90 days (due to potential long‐term viral shedding), we excluded all individuals with a positive SARS‐CoV‐2 test within 90 days before the observation period.^15^, ^20^ As the proportion of the BNT162b2 messenger RNA vaccine (Comirnaty, Biontech‐Pfizer) is more than 80% of all vaccine doses against SARS‐CoV‐2 ever used in Austria, we calculated the number of vaccine doses regardless of vaccine type.

From 1 November to 31 December 2022 we recorded COVID‐19 deaths, SARS‐CoV‐2 positive test results (regardless of symptoms), all‐cause mortality and vaccinations in the study population. Detection of SARS‐CoV‐2 infections was based on the EMS that only records polymerase chain reaction (PCR) tests or, restricted to the end of 2020 until May 2021, antigen tests from accredited diagnostic facilities. Presence of repeated previous documented infection was classified if there were > =2 positive test results more than 90 days apart. COVID‐19 deaths were classified in persons with a positive SARS‐CoV‐2 test who subsequently died due to COVID‐19 as recorded by the local public health office.

### 2.2 Statistical analysis

Categorical data are presented as percentages and continuous data are shown as medians (with 25th to 75th percentile). We calculated Cox proportional hazard ratios (HR) with 95% confidence intervals (CI) in groups according to the number of vaccine doses against SARS‐CoV‐2. We calculated age (untransformed continuous variable in years) and gender adjusted HR.^21^ We assumed that it takes 7 days for a SARS‐CoV‐2 vaccination to become effective.^6^ Thus, individuals changed their group allocation regarding vaccination status 7 days after receiving the vaccine dose. Sensitivity analyses changed group allocation at the day of vaccination and excluded all individuals who received any vaccine dose between 25 October and 24 December 2022.^22^ Censoring occurred at the end of the observation period, the date of non‐COVID19 death, the date of a fifth vaccination, or the date of the outcome event, whatever occurred first. Relative vaccine effectiveness (rVE) was calculated as (1‐HR) X 100.

Comparisons of groups with four versus three vaccinations were performed in the entire study population and in subgroups stratified by gender, age, presence or absence of repeated previous infections and year of the last previous infection. To evaluate potential waning immunity after vaccination, we performed analyses, stratified in those who received the fourth vaccine dose within 4 weeks, from >4–8 weeks, and >8 weeks before 1 November 2022. We performed additional analyses according to the presence or absence of repeated previous SARS‐CoV‐2 infections, according to the year of the last previous infection, and after exclusion of children and nursing home residents.

An extended observation period until 30 June 2023 was used to increase statistical power. These analyses were limited by unavailability of all‐cause mortality data during 2023, but the impact of this inaccuracy is negligible on our outcome analyses. Finally, analyses were performed for all‐cause mortality (limited to the last 2 months of 2022) with group allocation switching on the day of vaccination.^23^

Proportional hazards assumptions were checked by graphically examining Schoenfeld residuals and in case of violation it was planned to split the observation period. Cox proportional HR were only calculated for analyses with at least 10 outcome events. Statistical analyses were performed in R (version 4.2.2).

## 3 RESULTS

### 3.1 Study population

From the general population in Austria of 9,090,868 (as of October 1, 2022), 3,986,312 individuals were eligible (see Figure S1). Baseline characteristics are presented in Table 1 and the Supplements (Table S2), and there were no missing data. Main differences across the groups were that older individuals received more vaccinations. Number (%) of nursing home residents in the total cohort, and among those who received four, three, one or two, and no vaccination, were 30,286 (0.76), 15,479 (5.50), 10,264 (0.66), 2496 (0.27) and 2047 (0.17) respectively.

**TABLE 1:** Baseline characteristics of the entire study population as of November 1, 2022.

	All	Females	Males	Four vaccine doses	Three vaccine doses	One or two vaccine doses	Unvaccinated
Number	3,986,312	2047,454	1,938,858	281,291	1,545,242	933,277	1,225,317
Females	2047,454	2047,454	0	144,957	816,045	465,849	620,096
	(51·36%)	(100%)	(0%)	(51·53%)	(52·81%)	(49·92%)	(50·61%)
Age (years)	38 (22–54)	38 (23–54)	37 (21–53)	63 (49–75)	44 (30–57)	32 (20–47)	27 (11–44)
Five or more vaccine doses	1185	507	678	0	0	0	0
	(0·03%)	(0·02%)	(0·03%)	(0%)	(0%)	(0%)	(0%)
Four vaccine doses	281,291	144,957	136,334	281,291	0	0	0
	(7·06%)	(7·08%)	(7·03%)	(100%)	(0%)	(0%)	(0%)
Three vaccine doses	1,545,242	816,045	729,197	0	1,545,242	0	0
	(38·76%)	(39·85%)	(37·61%)	(0%)	(100%)	(0%)	(0%)
One or two vaccine doses	933,277	465,849	467,428	0	0	933,277	0
	(23·41%)	(22·75%)	(24·11%)	(0%)	(0%)	(100%)	(0%)
Unvaccinated	1,225,317	620,096	605,221	0	0	0	1,225,317
	(30·74%)	(30·29%)	(31·22%)	(0%)	(0%)	(0%)	(100%)
Time since last vaccination (days)	322 (279–345)	322 (278–345)	322 (279–344)	33 (19–69)	323 (290–340)	339 (308–403)	NA
Repeated previous infections	385,444	209,036	176,408	8771	75,694	117,590	183,362
	(9·67%)	(10·21%)	(9·10%)	(3·12%)	(4·90%)	(12·60%)	(14·96%)
Time since last infection (days)	247 (220–285)	245 (218–283)	250 (221–287)	236 (216–267)	232 (193–266)	264 (229–334)	265 (231–295)

*NOTE*: Data are n (%) or median (interquartile range). NA, not applicable.

### 3.2 COVID‐19 deaths and infections

During November and December 2022, we recorded 69 COVID‐19 deaths and 89,056 SARS‐CoV‐2 infections with an overall case fatality rate of 0.08%. Median (25th to 75th percentile) follow‐up time for the four vaccine dose group within this observation period was 51 days (27–60) for COVID‐19 mortality and 50 days (27–60) for SARS‐CoV‐2 infections. The Omicron sublineage BA.5 was the predominant variant with a significant increase of BQ.1 in December.

We did not observe significant differences of COVID‐19 deaths comparing groups with four versus three vaccine doses with a rVE of −24% (95% CI: −120 to 30), whereas there was a significant rVE with 17% (95% CI: 14–19) for SARS‐CoV‐2 infections (Table 2). There were no significant other group differences in COVID‐19 mortality, but fewer infections were recorded in the less vaccinated groups (Table 2).

**TABLE 2:** Cox proportional hazard ratios (HRs) with 95% confidence intervals (95% CIs) for COVID 19‐deaths and SARS‐CoV‐2 infections according to vaccination status from November 1 to December 31, 2022.

	Four vaccine doses	Three vaccine doses	One or two vaccine doses	Unvaccinated
	COVID‐19 deaths			
COVID‐19 deaths (*n*)	31	20	7	11
Events per 100,000 person days	0·10	0·02	0·01	0·01
Age and gender adjusted HR (95% CI)	1·24 (0·70–2·20)	Reference	1·19 (0·50–2·82)	1·56 (0·75–3·26)
	SARS‐CoV‐2 infections			
SARS‐CoV‐2 infections (*n*)	8511	37,624	22,554	20,367
Events per 100,000 person days	29·02	43·89	41·31	27·98
Age and gender adjusted HR (95% CI)	0·83 (0·81–0·86)	Reference	0·95 (0·93–0·96)	0·66 (0·65–0·67)

Subgroup analyses of four versus three vaccine doses were limited due to low number of COVID‐19 deaths (*n* = 51). Age and gender adjusted HR (with 95% CI) were 1.00 (0.54–1.87) in persons aged 75 years and older (42 events), 1.30 (0.56–2.99) in women (24 events), 1.21 (0.55–2.66) in men (27 events), 1.14 (0.63–2.06) in persons without repeated previous infections (47 events) and 1.06 (0.57–1.97) in persons with the last recorded previous infection in 2022 (43 events). No individual younger than 40 years died due to COVID‐19. Subgroup analyses on SARS‐CoV‐2 infections confirm a statistically significant rVE except for subgroups with the most recent previous infection before 2022 and individuals aged 75 years and older (Table S3).

For infections, rVE rapidly declined with time elapsed after the fourth vaccination (Table 3). Individuals with repeated previous infections had reduced re‐infection risk (Table S4). Analyses on infection rates according to year of the last prior infection showed significantly waning immunity with time elapsed since last infection (Table S5).

**TABLE 3:** Cox proportional hazard ratios (HRs) with 95% confidence intervals (95% CIs) for COVID‐19 deaths and SARS‐CoV‐2 infections from November 1 to December 31, 2022, for four versus three vaccine doses in subgroups according to time since the fourth vaccination.

	Study population with exclusion of newly vaccinated individuals from October 25 to December 24, 2022			
	Four vaccine doses	Four vaccine doses	Four vaccine doses	Three vaccine doses
	Time of fourth vaccination before November 1, 2022			
	within 4 weeks	> 4–8 weeks	> 8 weeks	All
	COVID‐19 deaths			
COVID‐19 deaths (*n*)	5	4	17	20
Events per 100,000 person days	0·07	0·10	0·33	0·03
Age and gender adjusted HR (95% CI)	0·77 (0·29–2·05)	0·80 (0·27–2·37)	1·53 (0·79–2·99)	Reference
	SARS‐CoV‐2 infections			
SARS‐CoV‐2 infections (*n*)	2101	1570	2744	36,927
Events per 100,000 person days	29·93	38·52	54·01	46·89
Age and gender adjusted (HR 95% CI)	0·66 (0·63–0·69)	0·86 (0·81–0·90)	1·22 (1·17–1·27)	Reference

### 3.3 Extended follow‐up

**FIGURE 1:**
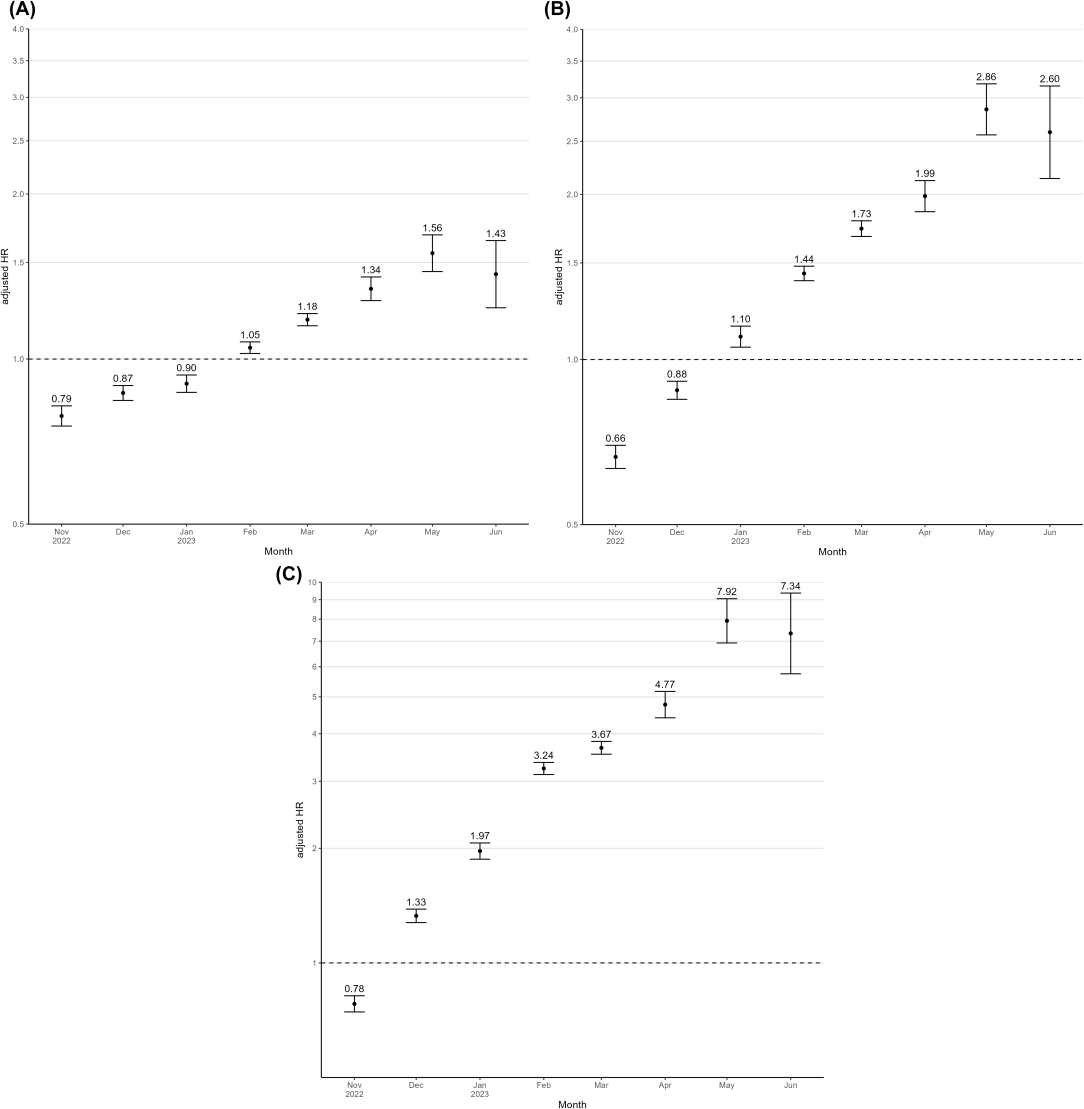
Age and gender adjusted hazard ratios (95% confidence intervals) of four versus less vaccine doses against SARS‐CoV‐2 infections shown for each month from November 2022 to June 2023. (A) Age and gender adjusted hazard ratios of four versus three vaccine doses. (B) Age and gender adjusted hazard ratios of four versus one or two vaccine doses. (C) Age and gender adjusted hazard ratios of four versus no vaccine dose.

On January 1, 2023, group sizes with four, three, one to two and no vaccination were, 490,623, 1,352,471, 911,896 and 1,223,216, respectively. Among those who had received four vaccine doses, the proportion of mRNA vaccines from Biontech‐Pfizer, mRNA vaccines from Moderna, and other vaccine types, were 97.4%, 2.4% and 0.2%, respectively. From 1 January to 30 June 2023, the sublineage XBB.1.5. was the predominant variant, and we recorded 225 COVID‐19 deaths and 174,174 SARS‐CoV‐2 infections. Analyses in 2023 confirm no rVE for four versus three vaccine doses for COVID‐19 mortality (4%, 95% CI: −31 to 29), but show higher risk of SARS‐CoV‐2 infections with a rVE of −17% (95% CI: −19 to −15) (Table 4). Until 30 June 2023, 536,376 individuals had received the fourth vaccine dose, with thus only relatively few additional fourth vaccinations in 2023. rVE of the fourth vaccination versus all less vaccinated groups gradually declined from November 2022 to June 2023 (Figure 1 and Table S6).

**TABLE 4:** Cox proportional hazard ratios (HRs) with 95% confidence intervals (95% CIs) for COVID 19‐deaths and SARS‐CoV‐2 infections according to vaccination status from 1 January to 30 June 2023.

	Four vaccine doses	Three vaccine doses	One or two vaccine doses	Unvaccinated
	COVID‐19 deaths			
COVID‐19 deaths (*n*)	95	75	26	29
Events per 100,000 person days	0·10	0·03	0·02	0·01
Age and gender adjusted HR (95% CI)	0·96 (0·71–1·31)	Reference	1·18 (0·76–1·85)	1·07 (0·70–1·65)
	SARS‐CoV‐2 infections			
SARS‐CoV‐2 infections (*n*)	29,808	80,246	39,156	24,964
Events per 100,000 person days	30·91	31·34	23·72	11·04
Age and gender adjusted HR (95% CI)	1·17 (1·15–1·19)	Reference	0·73 (0·72–0·74)	0·33 (0·33–0·34)

All analyses remained materially unchanged in sensitivity analyses with changing group allocation on the day of vaccination, by excluding individuals who received a vaccine dose between October 25 and December 24, 2022, or those who ever received non‐mRNA vaccines against SARS‐CoV‐2 (data not shown). Outcome analyses with exclusion of children did not significantly alter our results (Tables S7 to S9) nor did analyses with exclusion of nursing home residents (Tables S10 and S11). The majority of COVID‐19 deaths and a significant proportion of all‐cause deaths occurred in nursing home residents (Table S12). Proportional hazards assumptions appeared reasonable for most of our analyses, but we anyway applied a time‐axis division to present HR for each month separately to address this issue (Figure 1).

### 3.4 All‐cause mortality

In participants with four, three, one to two and no vaccinations, we recorded 1568, 1748, 607 and 566 all‐cause deaths from 1 November to 31 December 2022, respectively. Compared to individuals with three vaccinations, the age and gender adjusted HR (with 95% CI) for all‐cause mortality in those with four, one to two, and no vaccinations, was 0.79 (0.74–0.85), 1.17 (1.06–1.28) and 0.93 (0.85–1.02), respectively (see also Table S13). Excluding nursing home residents, age and gender adjusted HR (with 95% CI) for all‐cause mortality in those with four versus three vaccinations was 0.63 (0.57–0.69), while for nursing home residents it was 0.79 (0.71–0.89).

## 4 DISCUSSION

During the Omicron wave by the end of 2022 in Austria, individuals with a previous SARS‐CoV‐2 infection showed no significant difference in COVID‐19 mortality in groups receiving four versus three vaccine doses. For SARS‐CoV‐2 infections, we observed a small rVE of a fourth vaccine dose with evidence for rapidly waning immunity and reversal of this effect in 2023. Repeated previous and recent infections were both associated with reduced infection risk. All‐cause mortality data indicate modest healthy vaccinee bias.

Our findings on COVID‐19 mortality extend the few investigations on the effectiveness of a fourth vaccine dose on clinically significant outcomes in previously SARS‐CoV‐2 infected persons.^9^, ^10^, ^13^ A nationwide study from Italy showed that from 12 September to 11 December 2022 (i.e. 7 to 90 days after receiving the second booster), the rVE of a second bivalent mRNA booster dose versus a first monovalent mRNA booster was approximately 62% in reducing severe COVID‐19 in persons ≥60 years with previous infection.^9^ A study from the US during mid‐2022 reported that in adults aged ≥50 years with previous SARS‐CoV‐2 infection, rVE of four versus three mRNA‐1273 vaccines was 34.8% (95% CI: 26.5–42.1) for a combined outcome (SARS‐CoV‐2 infections, COVID‐19 hospitalizations and COVID‐19 hospital deaths).^10^ A study in adults from Singapore between 14 October 2022 and 31 January 2023, showed that in previously infected individuals, a fourth bivalent vaccine dose reduced symptomatic SARS‐CoV‐2 infections and COVID‐19‐related hospital admissions by 86% and 96%, respectively, during 2 months after vaccination.^13^ These studies had significantly shorter follow‐up times after vaccination than our study, and used combined endpoints including hospitalized patients with SARS‐CoV‐2 to classify severe COVID‐19. Such classifications for severe COVID‐19 must be interpreted with caution, because during Omicron waves, many hospitalized patients with positive SARS‐CoV‐2 tests presented with mild symptoms or were even asymptomatic and detected only because of routine admission screening.^2^, ^18^, ^24^ Thus, there is a need for investigations on vaccine effectiveness that assess also COVID‐19 mortality, as in our study, to disentangle rVE for hard clinical outcomes versus positive laboratory tests for SARS‐CoV‐2 with unclear and probably no adverse consequences for most individuals (even for hospitalized patients) in an endemic phase.^2^, ^18^, ^24^

Our results on a significant rVE of four versus three vaccine doses with regard to laboratory confirmed SARS‐CoV‐2 infections corroborate findings from several other studies.^5^, ^8^, ^12^, ^22^, ^25^ Estimates of rVE were larger in previous studies, but this may be due, at least partly, to shorter follow‐up.^5^, ^8^, ^12^, ^22^, ^25^ Evidence of peak effectiveness about 3–5 weeks after receiving the fourth vaccine dose, but then decreasing effectiveness towards no remaining effect beyond 15 weeks was previously reported and fits well to our findings.^5^, ^25^ The public health significance of this transient risk reduction in SARS‐CoV‐2 infections lasting for several weeks after receiving the fourth vaccine dose remains unclear. Although this reduced infection risk did not translate into prevention of COVID‐19 deaths according to our data, we cannot exclude other benefits related to non‐fatal adverse health outcomes following SARS‐CoV‐2 infections. While rapidly waning vaccine protection is observed for laboratory confirmed SARS‐CoV‐2 infections, previous studies documented that vaccine effectiveness seems to be long lasting for protection against severe and lethal COVID‐19.^5^, ^17^ Similarly, data from Qatar suggest that natural immunity confers a very strong protection against severe COVID‐19 with no evidence of waning immunity, a conclusion that is supported by a systematic review and meta‐analyses.^14^, ^16^ Thus, SARS‐CoV‐2 infections and/or vaccinations, have contributed to the transition of this COVID‐19 pandemic into endemicity with very low case fatality rates, as documented in our investigation.^2^, ^14^ The relative contribution to this protection against COVID‐19 mortality by natural and/or vaccine induced immunity, by the characteristics of the Omicron variant, by advances in COVID‐19 therapy or by other factors, remains speculative.

Consistent with the literature on waning natural immunity, we observed increasing risk of SARS‐CoV‐2 infections with time elapsed after the last prior infection. As not only time but also virus variants may underlie the observed declining immunity after previous infections, we note that the end of 2021 and beginning of 2022 marked the beginning of the Omicron wave in Austria. The magnitude of the changes in infection risk as a function of time elapsed after the last previous infection suggest that natural immunity may be a main determinant of immunological protection in a population (Table S3).

Compared to three vaccine doses, those with fewer or no vaccinations did not differ with regard to COVID‐19 mortality but had reduced risk of SARS‐CoV‐2 infections. Of note, less vaccinated groups yielded also significantly lower SARS‐CoV‐2 infection risk compared to the four vaccine dose group in 2023, a finding that fits well to a relatively long‐term follow‐up study from Qatar.^26^ In that study, comparing the third versus the second vaccination, the rVE for SARS‐CoV‐2 infections was highest with 61.4% (95% CI: 60.2–62.6) in the first month of follow‐up and gradually declined to a negative rVE with −45.7% (95% CI: approximately −60 to −30) after 11 months follow‐up.^26^ It was hypothesized that immune imprinting might explain this effect, as prior exposure to a primary antigen (e.g. the ancestral SARS‐CoV‐2 vaccine) can attenuate the immunity against subsequent infections (or vaccinations) of related but novel antigens (e.g. new virus variants), because the immune response is skewed towards the ancestral antigen.^26^, ^27^, ^28^ Compared to unvaccinated controls, compromised humoral immunity against SARS‐CoV‐2 variants of concern (e.g. Omicron) in triple vaccinated humans and animals has been documented, and may, at least in part, explain our findings.^27^, ^28^ To what extent other factors such as a hypothetically reduced willingness to test for SARS‐CoV‐2 in those who refuse vaccinations, bias, or other factors (e.g. stronger infection derived immunity) may explain the particularly low infection risk in unvaccinated or less vaccinated persons, remains speculative. We consider the higher prevalence of repeated previous infections in these less vaccinated individuals to be consistent with protective effects of vaccinations during the course of this pandemic.

We observed a 21% lower all‐cause mortality risk in individuals with four versus three vaccine doses (37% when we excluded nursing home residents), suggesting healthy vaccinee bias especially in the community‐dwelling population, albeit much lower as in a study from Israel.^23^ In that study, individuals who received three versus two vaccinations had a 94.6% lower risk of COVID‐19 deaths, but also a 94.8% lower risk of non‐COVID‐19 mortality.^23^ Healthy vaccinee bias may cause overestimation of rVE, but this would not materially alter our findings, as we have not observed a protective effect for COVID‐19 deaths anyhow. As huge differences in all‐cause mortality are likewise also paralleled by differences in hospitalizations, this might also affect other endpoints such as severe COVID‐19 (that includes hospitalizations). On the other hand, group differences in all‐cause mortality may have an impact on COVID‐19 outcomes, for example, by competing risks, as someone who died due to non‐COVID‐19 diseases cannot die due to COVID‐19 anymore. Moreover, there can be some misclassification between COVID‐19 and non‐COVID‐19 deaths.^29^

Our findings are limited due to the observational design that precludes definite conclusions regarding causality. In general, observational studies of COVID‐19 vaccine effectiveness are subject to multiple possible biases.^30^, ^31^ The low number of COVID‐19 deaths warrant caution regarding data interpretation and we also have to note the unequally sized groups and the relatively long time elapsed after the last vaccination in individuals who received three vaccine doses. However, a long lapse with waning immune protection would suggest better chances, if anything, for showing benefits from a 4th dose. We have to acknowledge the strong dependence on the data quality of the EMS with subsequent potential sources of bias and/or confounding. These include among others, limitations regarding reporting of data, access and indications for SARS‐CoV‐2 tests with missing data on testing frequencies and persons who moved away from Austria during the study, potential behaviour changes in response to vaccination and/or SARS‐CoV‐2 infection, and test results, as well as test accuracy that may all vary over time. We did not have access to data regarding co‐morbidities and medications and could therefore not adjust for them. While we had no detailed data on co‐morbidities, we could use national data on nursing home residency, which represents a surrogate for many co‐morbidities and the strongest factor that may affect infection fatality rate.^32^ Thus, we could also perform analyses excluding nursing home residents that give fair estimates of rVE in the free, community‐living general population. These sensitivity analyses yielded similar results and therefore strengthen our findings. Subgroup analyses according to age did also not materially change our findings. Unavailability of data on monovalent versus bivalent vaccinations precluded separate analyses of these two different vaccine types. Bivalent vaccines were mainly recommended in Austria from mid‐September 2022 on and were thus likewise the predominant vaccine received as the fourth vaccination in our study. Of note, superior effectiveness of bivalent versus monovalent mRNA vaccines against SARS‐CoV‐2 has been documented.^11^, ^13^ The lack of effectiveness of the fourth vaccination during 2023 in our study is, however, consistent with the notion of rapidly waning immunity by this second, mainly bivalent, booster. Finally, our findings do not apply to previously uninfected individuals, a population group that is vanishingly small by late 2023.

In conclusion, in the general population of Austria with a history of a SARS‐CoV‐2 infection we did not observe a significant rVE of a fourth vaccine dose for COVID‐19 deaths during a time with already very low absolute risk for this outcome. We documented a transient rVE for SARS‐CoV‐2 infections, but this effect was reversed during extended follow‐up in 2023. Repeated previous and more recent SARS‐CoV‐2 infections were both associated with significantly reduced reinfections. In general, our study results question whether recommendations for repeated vaccine boosters against SARS‐CoV‐2 are currently justified for large parts of the general population with a history of previous infections. This does not contradict the health benefit of the initial vaccinations of unprotected populations in the early phase of the COVID‐19‐pandemic and of vaccinations of very high‐risk populations at any time.^4^ Our findings fit well to the hypothesis of diminishing effectiveness and thus shifting risk–benefit ratios from additional vaccinations during the transition of the COVID‐19 pandemic to its endemic phase.^2^ In view of the strong and population‐wide immunological protection due to previous infections and vaccinations, it is tempting to speculate that SARS‐CoV‐2 infections may already resemble by 2023 other human coronaviruses.^2^ Our data require confirmation in other national populations and are important to inform future public health and vaccine policy regarding COVID‐19, but also underscore the critical role of active national health surveillance during a pandemic.

## AUTHOR CONTRIBUTIONS

*Concept and design*: Stefan Pilz and John PA Ioannidis. *Drafting of the manuscript*: Stefan Pilz and John PA Ioannidis. *Statistical analysis*: Alena Chalupka and Lukas Richter. Acquisition or interpretation of data: All authors Critical review of the manuscript for important intellectual content: All authors.

## FUNDING INFORMATION

This work is funded by the Austrian Science Fund (FWF) KLI 1188.

## CONFLICT OF INTEREST STATEMENT

None.

## Supporting information


**Data S1.** Supporting Information
