# Supplementary material for: Effectiveness of a fourth SARS‐CoV‐2 vaccine dose in previously infected individuals from Austria
Source: Eur J Clin Invest. 2023 Nov 30;54(3):e14136. doi: 10.1111/eci.14136 (PMC11475503; doi:10.1111/eci.14136)
Supplement: Supplementary file 1 — Data S1. Supporting Information [file ECI-54-e14136-s001.docx]

**Table of Contents Page**

**Table S1: STROBE checklist for cohort studies………………………………………………………………...2**

**Table S2: Additional baseline characteristics…………………………………………………………………...4**

**Table S3: Cox proportional hazard ratios for SARS-CoV-2 infections in subgroup analyses……………….5**

**Table S4: Cox proportional hazard ratios for SARS-CoV-2 infections for individuals with and without previous SARS-CoV-2 infections according to number of vaccinations………………………………………6**

**Table S5: Cox proportional hazard ratios for SARS-CoV-2 infections for individuals according to the year of the last previous SARS-CoV-2 infections according to number of vaccinations…………...………………7**

**Table S6: Cox proportional hazard ratios (HRs) with 95% confidence intervals (95% CIs) for SARS-CoV-2 infections according to each month from November 2022 to June 2023 comparing the group with four vaccinations versus groups with three, one to two and no vaccination………………………………………..8**

**Table S7: Cox proportional hazard ratios (HRs) with 95% confidence intervals (95% CIs) for COVID 19-deaths and SARS-CoV-2 infections according to vaccination status from November 1 to December 31, 2022 in adults (all individuals aged at least 19 years)…………………………………………………………..9**

**Table S8: Cox proportional hazard ratios (HRs) with 95% confidence intervals (95% CIs) for COVID 19-deaths and SARS-CoV-2 infections according to vaccination status from January 1 to June 30, 2023 in adults (all individuals aged at least 19 years)………………………………………………………………….10**

**Table S9: Cox proportional hazard ratios (HRs) with 95% confidence intervals (95% CIs) for SARS-CoV-2 infections according to each month from November 2022 to June 2023, comparing the group with four vaccinations versus groups with three, one to two and no vaccination in adults (all individuals aged at least 19 years)………………...………………………………………………………………………………………...11**

**Table S10: Cox proportional hazard ratios (HRs) with 95% confidence intervals (95% CIs) for COVID 19-deaths and SARS-CoV-2 infections according to vaccination status from November 1 to December 31, 2022, in the entire study cohort excluding nursing home residents…………………..………………………12**

**Table S11: Cox proportional hazard ratios (HRs) with 95% confidence intervals (95% CIs) for all-cause mortality according to vaccination status from November 1 to December 31, 2022, in the entire study cohort excluding nursing home residents………………………………………………………………………13**

**Table S12: COVID-19 deaths and all-cause deaths according to vaccination status and nursing home residence status from November 1 to December 31, 2022, in the entire study cohort………………………14**

**Table S13: Cox proportional hazard ratios (HRs) with 95% confidence intervals (95% CIs) for all-cause deaths in November 2022 and December 2022, comparing the group with four vaccinations versus groups with three, one to two and no vaccination……………………………………………………………………...15**

**Figure S1: Participant selection chart………………………………………………………………………….16**

**Table S1: STROBE Statement—Checklist of items for *cohort studies***

|  | **Item No** | **Recommendation** | **Main text page** |  |  |
| --- | --- | --- | --- | --- | --- |
| **Title and abstract** | 1 | (*a*) Indicate the study’s design with a commonly used term in the title or the abstract | Page 2, Abstract |  |  |
|  |  | (*b*) Provide in the abstract an informative and balanced summary of what was done and what was found | Page 2 and 3, Abstract |  |  |
| **Introduction** | | |  |  |  |
| Background/rationale | 2 | Explain the scientific background and rationale for the investigation being reported | Page 4, Introduction |  |  |
| Objectives | 3 | State specific objectives, including any prespecified hypotheses | Page 4 and 5, Introduction |  |  |
| **Methods** | | |  |  |  |
| Study design | 4 | Present key elements of study design early in the paper | Page 5, Methods |  |  |
| Setting | 5 | Describe the setting, locations, and relevant dates, including periods of recruitment, exposure, follow-up, and data collection | Page 5 and 6, Methods |  |  |
| Participants | 6 | (*a*) Give the eligibility criteria, and the sources and methods of selection of participants. Describe methods of follow-up | Page 6, Methods |  |  |
|  |  | (*b*) For matched studies, give matching criteria and number of exposed and unexposed | Not applicable |  |  |
| Variables | 7 | Clearly define all outcomes, exposures, predictors, potential confounders, and effect modifiers. Give diagnostic criteria, if applicable | Page 5 to 7, Introduction and Methods |  |  |
| Data sources/ measurement | 8* | For each variable of interest, give sources of data and details of methods of assessment (measurement). Describe comparability of assessment methods if there is more than one group | Page 5 and 6, Methods |  |  |
| Bias | 9 | Describe any efforts to address potential sources of bias | Page 6 and 7, national cohort, adjusted statistical analyses |  |  |
| Study size | 10 | Explain how the study size was arrived at | Page 5, Methods, no sample size calculation |  |  |
| Quantitative variables | 11 | Explain how quantitative variables were handled in the analyses. If applicable, describe which groupings were chosen and why | Page 6, Methods/Statistical Analysis |  |  |
| Statistical methods | 12 | (*a*) Describe all statistical methods, including those used to control for confounding | Page 6 to 7, Methods/Statistical Analysis |  |  |
|  |  | (*b*) Describe any methods used to examine subgroups and interactions | Page 6 to 7, Methods/Statistical Analysis |  |  |
|  |  | (*c*) Explain how missing data were addressed | Page 8, Results, no missing data |  |  |
|  |  | (*d*) If applicable, explain how loss to follow-up was addressed | Not applicable |  |  |
|  |  | (*e*) Describe any sensitivity analyses | Page 7, Methods, Statistical Analysis |  |  |
| **Results** | | |  |  |  |
| Participants | 13* | (a) Report numbers of individuals at each stage of study—eg numbers potentially eligible, examined for eligibility, confirmed eligible, included in the study, completing follow-up, and analysed | Page 8 and 10, Results; Table 1 |  |  |
|  |  | (b) Give reasons for non-participation at each stage | Page 6, Methods and Page 8, Results |  |  |
|  |  | (c) Consider use of a flow diagram | Supplements eFigure 1 |  |  |
| Descriptive data | 14* | (a) Give characteristics of study participants (eg demographic, clinical, social) and information on exposures and potential confounders | Table 1, Supplements eTable 2 |  |  |
|  |  | (b) Indicate number of participants with missing data for each variable of interest | Page 8, Results, no missing data |  |  |
|  |  | (c) Summarise follow-up time (eg, average and total amount) | Page 8, Results |  |  |
| Outcome data | 15* | Report numbers of outcome events or summary measures over time | Page 8 and 10, Results; Tables 2 and 4 |  |  |
| Main results | 16 | (*a*) Give unadjusted estimates and, if applicable, confounder-adjusted estimates and their precision (eg, 95% confidence interval). Make clear which confounders were adjusted for and why they were included | Page 8 to 10, Results; Tables 2 to 4 |  |  |
|  |  | (*b*) Report category boundaries when continuous variables were categorized | Page 8 to 10, Results; Tables 2 to 4 |  |  |
|  |  | (*c*) If relevant, consider translating estimates of relative risk into absolute risk for a meaningful time period | Page 8 and 9, Results |  |  |
| Other analyses | 17 | Report other analyses done—eg analyses of subgroups and interactions, and sensitivity analyses | Page 8 to 10, Results; Supplements eTables 2 to 13 |  |  |
| **Discussion** | | |  |  |  |
| Key results | 18 | Summarise key results with reference to study objectives | Page 10 and 14, Discussion |  |  |
| Limitations | 19 | Discuss limitations of the study, taking into account sources of potential bias or imprecision. Discuss both direction and magnitude of any potential bias | Page 13, Discussion |  |  |
| Interpretation | 20 | Give a cautious overall interpretation of results considering objectives, limitations, multiplicity of analyses, results from similar studies, and other relevant evidence | Page 14, Discussion |  |  |
| Generalisability | 21 | Discuss the generalisability (external validity) of the study results | This is a nationwide survey, Page 5 |  |  |
| **Other information** | | |  |  |  |
| Funding | 22 | Give the source of funding and the role of the funders for the present study and, if applicable, for the original study on which the present article is based | Page 15 |  |  |

*Give information separately for exposed and unexposed groups.

**Note:** An Explanation and Elaboration article discusses each checklist item and gives methodological background and published examples of transparent reporting. The STROBE checklist is best used in conjunction with this article (freely available on the Web sites of PLoS Medicine at http://www.plosmedicine.org/, Annals of Internal Medicine at http://www.annals.org/, and Epidemiology at http://www.epidem.com/). Information on the STROBE Initiative is available at http://www.strobe-statement.org.

| **Table S2: Additional Baseline characteristics of the entire study population as of November 1, 2022** | | | | | | | |
| --- | --- | --- | --- | --- | --- | --- | --- |
|  | ≥ 75 years | 60 to < 75 years | 40 to < 60 years | 19 to < 40 years | < 19 years | Single previous infection | Repeated previous infections |
| Number | 201,474 | 456,543 | 1,210,306 | 1,312,594 | 805,395 | 3,600,868 | 385,444 |
| Females | 114,667 | 233,531 | 634,882 | 671,789 | 392,585 | 1,838,418 | 209,036 |
|  | (56.91%) | (51.15%) | (52.46%) | (51.18%) | (48.74%) | (51.05%) | (54.23%) |
| Age (years) | 81 (78 - 85) | 65 (62 - 69) | 49 (44 - 54) | 30 (25 - 35) | 11 (8 - 15) | 38 (22 - 54) | 33 (21 - 47) |
| Five or more vaccine doses | 353 | 364 | 337 | 124 | 7 | 1,158 | 27 |
|  | (0.18%) | (0.08%) | (0.03%) | (0.01%) | (0.00%) | (0.03%) | (0.01%) |
| Four vaccine doses | 75,622 | 89,146 | 76,921 | 36,106 | 3,496 | 272,520 | 8,771 |
|  | (37.53%) | (19.53%) | (6.36%) | (2.75%) | (0.43%) | (7.57%) | (2.28%) |
| Three vaccine doses | 86,591 | 224,159 | 589,794 | 549,718 | 94,980 | 1,469,548 | 75,694 |
|  | (42.98%) | (49.10%) | (48.73%) | (41.88%) | (11.79%) | (40.81%) | (19.64%) |
| One or two vaccine doses | 19,238 | 61,905 | 258,436 | 386,909 | 206,789 | 815,687 | 117,590 |
|  | (9.55%) | (13.56%) | (21.35%) | (29.48%) | (25.68%) | (22.65%) | (30.51%) |
| Unvaccinated | 19,670 | 80,969 | 284,818 | 339,737 | 500,123 | 1,041,955 | 183,362 |
|  | (9.76%) | (17.74%) | (23.53%) | (25.88%) | (62.10%) | (28.94%) | (47.57%) |
| Time since last vaccination (days) | 258 (63 - 348) | 322 (118 - 342) | 327 (291 - 346) | 323 (290 - 346) | 303 (271 - 335) | 322 (278 - 344) | 329 (287 - 358) |
| Repeated previous infections | 7,820 | 22,013 | 117,158 | 158,768 | 79,685 | 0 | 385,444 |
|  | (3.88%) | (4.82%) | (9.68%) | (12.10%) | (9.89%) | (0%) | (100%) |
| Time since last infection (days) | 235 (202 - 292) | 239 (209 - 286) | 246 (217 - 285) | 249 (220 - 285) | 255 (228 - 284) | 251 (222 - 289) | 228 (139 - 258) |
| Most recent infection | 2020 | 2021 | 2022 |  |  |  |  |
| Number | 152,967 | 617,324 | 3,216,021 |  |  |  |  |
| Females | 76,814 | 301,193 | 1,669,447 |  |  |  |  |
|  | (50.22%) | (48.79%) | (51.91%) |  |  |  |  |
| Age (years) | 49 (31 - 61) | 38 (21 - 54) | 37 (22 - 53) |  |  |  |  |
| Five or more vaccine doses | 79 | 100 | 1,006 |  |  |  |  |
|  | (0.05%) | (0.02%) | (0.03%) |  |  |  |  |
| Four vaccine doses | 14,920 | 24,730 | 241,641 |  |  |  |  |
|  | (9.75%) | (4.01%) | (7.51%) |  |  |  |  |
| Three vaccine doses | 62,275 | 134,710 | 1,348,257 |  |  |  |  |
|  | (40.71%) | (21.82%) | (41.92%) |  |  |  |  |
| One or two vaccine doses | 59,344 | 191,734 | 682,199 |  |  |  |  |
|  | (38.80%) | (31.06%) | (21.21%) |  |  |  |  |
| Unvaccinated | 16,349 | 266,050 | 942,918 |  |  |  |  |
|  | (10.69%) | (43.10%) | (29.32%) |  |  |  |  |
| Time since last vaccination (days) | 313 (265 - 337) | 293 (192 - 353) | 325 (287 - 344) |  |  |  |  |
| Repeated previous infections | 19 | 8,989 | 376,436 |  |  |  |  |
|  | (0.01%) | (1.46%) | (11.71%) |  |  |  |  |
| Time since last infection (days) | 715 (699 - 727) | 361 (343 - 546) | 236 (210 - 264) |  |  |  |  |
| Data are n (%) or median (interquartile range) | | | | | | | |

| **Table S3: Cox proportional hazard ratios (HRs) with 95% confidence intervals (95% CIs) for SARS-CoV-2 infections from November 1 to December 31, 2022, for four versus three vaccine doses in subgroups according to age, gender, presence or absence of repeated previous SARS-CoV-2 infections, and year of the last previous SARS-CoV-2 infection** | | | | | | |
| --- | --- | --- | --- | --- | --- | --- |
|  | Four vaccine doses | Three vaccine doses | Four vaccine doses | Three vaccine doses | Four vaccine doses | Three vaccine doses |
|  | Age groups | | | | | |
|  | ≥75 years | | 60 to <75 years | | 40 to < 60 years | |
| SARS-CoV-2 infections (n) | 1,795 | 1,369 | 2,039 | 3,481 | 2,905 | 15,911 |
| Events per 100,000 person days | 29.97 | 31.22 | 23.17 | 29.77 | 30.94 | 48.58 |
| Age and gender adjusted HR (95% CI) | 0.99 (0.92 - 1.06) | Reference | 0.92 (0.87 - 0.98) | Reference | 0.81 (0.78 - 0.84) | Reference |
|  | Age groups | | | |  |  |
|  | 19 to < 40 years | | < 19 years | | Females | |
| SARS-CoV-2 infections (n) | 1,722 | 15,333 | 50 | 1,530 | 4,854 | 22,953 |
| Events per 100,000 person days | 35.36 | 48.71 | 12.63 | 26.45 | 31.76 | 50.84 |
| Age and gender adjusted HR (95% CI) | 0.91 (0.86 - 0.95) | Reference | 0.58 (0.44 - 0.77) | Reference | 0.79 (0.77 - 0.82) | Reference |
|  |  |  | Repeated previous infections | | | |
|  | Males | | Yes | | No | |
| SARS-CoV-2 infections (n) | 3,657 | 14,671 | 247 | 1,611 | 8,264 | 36,013 |
| Events per 100,000 person days | 26.03 | 36.15 | 23.98 | 36.63 | 29.20 | 44.28 |
| Age and gender adjusted HR (95% CI) | 0.89 (0.86 - 0.93) | Reference | 0.82 (0.71 - 0.94) | Reference | 0.83 (0.81 - 0.86) | Reference |
|  | Most recent SARS-CoV-2 infection | | | | | |
|  | 2020 | | 2021 | | 2022 | |
| SARS-CoV-2 infections (n) | 917 | 2,984 | 1,035 | 4,897 | 6,559 | 29,743 |
| Events per 100,000 person days | 68.19 | 84.14 | 45.85 | 63.00 | 25.49 | 39.97 |
| Age and gender adjusted HR (95% CI) | 0.98 (0.90 - 1.06) | Reference | 0.93 (0.87 - 1.00) | Reference | 0.83 (0.80 - 0.85) | Reference |

| **Table S4: Cox proportional hazard ratios (HRs) with 95% confidence intervals (95% CIs) for SARS-CoV-2 infections from November 1 to December 31, 2022, for individuals with and without repeated previous SARS-CoV-2 infections stratified according to the number of vaccinations** | | | | |
| --- | --- | --- | --- | --- |
|  | Repeated previous SARS-CoV-2 infections | | | |
|  | Yes | No | Yes | No |
|  | Four vaccine doses | | Three vaccine doses | |
| SARS-CoV-2 infections (n) | 247 | 8,264 | 1,611 | 36,013 |
| Events per 100,000 person days | 23.98 | 29.20 | 36.63 | 44.28 |
| Age and gender adjusted HR (95% CI) | 0.84 (0.74 - 0.95) | Reference | 0.82 (0.78 - 0.86) | Reference |
| Age, gender and year of the last previous infection adjusted HR (95% CI) | 0.94 (0.82 - 1.06) | Reference | 0.89 (0.85 - 0.94) | Reference |
|  | One or two vaccine doses | | Unvaccinated | |
|  | Yes | No | Yes | No |
| SARS-CoV-2 infections (n) | 2,300 | 20,254 | 2,035 | 18,332 |
| Events per 100,000 person days | 33.50 | 42.43 | 18.66 | 29.63 |
| Age and gender adjusted HR (95% CI) | 0.78 (0.74 - 0.81) | Reference | 0.62 (0.59 - 0.65) | Reference |
| Age, gender and year of the last previous infection adjusted HR (95% CI) | 0.96 (0.92 -1.00) | Reference | 0.63 (0.60 - 0.66) | Reference |

| **Table S5: Cox proportional hazard ratios (HRs) with 95% confidence intervals (95% CIs) for SARS-CoV-2 infections from November 1 to December 31, 2022, for individuals according to the year of the most recent previous SARS-CoV-2 infection and stratified according to the number of vaccinations** | | | | | | |
| --- | --- | --- | --- | --- | --- | --- |
|  | Year of the last previous SARS-CoV-2 infection | | | | | |
|  | 2020 | 2021 | 2022 | 2020 | 2021 | 2022 |
|  | Four vaccine doses | | | Three vaccine doses | | |
| SARS-CoV-2 infections (n) | 917 | 1,035 | 6,559 | 2,984 | 4,897 | 29,743 |
| Events per 100,000 person days | 68·19 | 45·85 | 25·49 | 84·14 | 63·00 | 39·97 |
| Age and gender adjusted HR (95% CI) | Reference | 0·67 | 0·37 | Reference | 0·74 | 0·45 |
|  |  | (0·61 - 0·73) | (0·34 - 0·40) |  | (0·71 - 0·77) | (0·43 - 0·47) |
| Age, gender and presence of repeated previous infections adjusted HR (95% CI) | Reference | 0·67 | 0·37 | Reference | 0·74 | 0·45 |
|  |  | (0·61 - 0·73) | (0·35 - 0·40) |  | (0·70 - 0·77) | (0·44 - 0·47) |
|  | One or two vaccine doses | | | Unvaccinated | | |
| SARS-CoV-2 infections (n) | 2,849 | 6,214 | 13,491 | 489 | 4,818 | 15,060 |
| Events per 100,000 person days | 84·44 | 55·75 | 33·66 | 50·69 | 30·50 | 26·88 |
| Age and gender adjusted HR (95% CI) | Reference | 0·67 | 0·41 | Reference | 0·66 | 0·59 |
|  |  | (0·64 - 0·70) | (0·39 - 0·43) |  | (0·60 - 0·73) | (0·54 - 0·64) |
| Age, gender and presence of repeated previous infections adjusted HR (95% CI) | Reference | 0·67 | 0·41 | Reference | 0·65 | 0·63 |
|  |  | (0·64 - 0·70) | (0·40 - 0·43) |  | (0·59 - 0·72) | (0·58 - 0·70) |

| **Table S6: Cox proportional hazard ratios (HRs) with 95% confidence intervals (95% CI) for SARS-CoV-2 infections according to each month from November 2022 to June 2023, comparing the group with four vaccinations versus groups with three, one to two and no vaccination** | | | | | | | | |
| --- | --- | --- | --- | --- | --- | --- | --- | --- |
|  | November 2022 | December 2022 | January 2023 | February 2023 | March 2023 | April 2023 | May 2023 | June 2023 |
|  | Age and gender adjusted HRs (95% CIs) for the group with four vaccine doses as compared to the respective reference group | | | | | | | |
| Three vaccine doses (reference) | 0.79 | 0.87 | 0.90 | 1.05 | 1.18 | 1.34 | 1.56 | 1.43 |
|  | (0.75-0.82) | (0.84-0.89) | (0.87-0.94) | (1.02-1.07) | (1.15-1.21) | (1.28-1.41) | (1.44-1.68) | (1.24-1.64) |
| One or two vaccine doses (reference) | 0.66 | 0.88 | 1.10 | 1.44 | 1.73 | 1.99 | 2.86 | 2.60 |
|  | (0.63-0.70 | (0.85-0.91) | (1.05-1.15) | (1.39-1.48) | (1.68-1.79) | (1.86-2.12) | (2.57-3.18) | (2.14-3.15) |
| Unvaccinated (reference) | 0.78 | 1,33 | 1.97 | 3.24 | 3.67 | 4.77 | 7.92 | 7.34 |
|  | (0.74-0.82) | (1,28-1,39) | (1.87-2.07) | (3.13-3.36) | (3.53-3.82) | (4.41-5.17) | (6.93-9.06) | (5.75-9.37) |

| **Table S7: Cox proportional hazard ratios (HRs) with 95% confidence intervals (95% CIs) for COVID 19-deaths and SARS-CoV-2 infections according to vaccination status from November 1 to December 31, 2022 in adults (all individuals aged at least 19 years)** | | | | |
| --- | --- | --- | --- | --- |
|  | Four vaccine doses | Three vaccine doses | One or two vaccine doses | Unvaccinated |
|  | COVID-19 deaths | | | |
| COVID-19 deaths (n) | 31 | 20 | 7 | 11 |
| Events per 100,000 person days | 0.11 | 0.02 | 0.02 | 0.03 |
| Age and gender adjusted HR (95% CI) | 1.24 (0.70 - 2.20) | Reference | 1.19 (0.50 - 2.82) | 1.56 (0.75 - 3.26) |
|  | SARS-CoV-2 infections | | | |
| SARS-CoV-2 infections (n) | 8,461 | 36,094 | 18,965 | 14,445 |
| Events per 100,000 person days | 29.24 | 45.09 | 44.46 | 33.51 |
| Age and gender adjusted HR (95% CI) | 0.85 (0.83 - 0.87) | Reference | 0.97 (0.95 - 0.98) | 0.73 (0.71 - 0.74) |

| **Table S8: Cox proportional hazard ratios (HRs) with 95% confidence intervals (95% CIs) for COVID 19-deaths and SARS-CoV-2 infections according to vaccination status from January 1 to June 30, 2023 in adults (all individuals aged at least 19 years)** | | | | |
| --- | --- | --- | --- | --- |
|  | Four vaccine doses | Three vaccine doses | One or two vaccine doses | Unvaccinated |
|  | COVID-19 deaths | | | |
| COVID-19 deaths (n) | 95 | 75 | 26 | 29 |
| Events per 100,000 person days | 0.10 | 0.03 | 0.02 | 0.02 |
| Age and gender adjusted HR (95% CI) | 0.96 (0.71 - 1.31) | Reference | 1.18 (0.76 - 1.85) | 1.08 (0.70 - 1.65) |
|  | SARS-CoV-2 infections | | | |
| SARS-CoV-2 infections (n) | 29,488 | 75,813 | 31,391 | 16,554 |
| Events per 100,000 person days | 31.01 | 31.54 | 23.98 | 12.22 |
| Age and gender adjusted HR (95% CI) | 1.18 (1.17 - 1.20) | Reference | 0.74 (0.73 - 0.75) | 0.38 (0.37 - 0.39) |

| **Table S9: Cox proportional hazard ratios (HRs) with 95% confidence intervals (95% CIs) for SARS-CoV-2 infections according to each month from November 2022 to June 2023, comparing the group with four vaccinations versus groups with three, one to two and no vaccination in adults (all individuals aged at least 19 years)** | | | | | | | | |
| --- | --- | --- | --- | --- | --- | --- | --- | --- |
|  | November 2022 | December 2022 | January 2023 | February 2023 | March 2023 | April 2023 | May 2023 | June 2023 |
|  | Age and gender adjusted HRs (95% CIs) for the group with four vaccine doses as compared to the respective reference group | | | | | | | |
| Three vaccine doses (reference) | 0.81 | 0.88 | 0.92 | 1.07 | 1.19 | 1.36 | 1.57 | 1.44 |
|  | (0.77-0.84) | (0.86-0.91) | (0.88-0.95) | (1.04-1.09) | (1.16-1.22) | (1.30-1.43) | (1.45-1.69) | (1.25-1.65) |
| One or two vaccine doses (reference) | 0.69 | 0.90 | 1.11 | 1.45 | 1.73 | 1.97 | 2.72 | 2.49 |
|  | (0.66-0.72) | (0.87-0.94) | (1.06-1.16) | (1.41-1.50) | (1.67-1.79) | (1.85-2.10) | (2.45-3.03) | (2.06-3.01) |
| Unvaccinated (reference) | 0.85 | 1.37 | 1.98 | 3.03 | 3.42 | 4.23 | 6.56 | 5.86 |
|  | (0.81-0.89) | (1.32-1.43) | (1.89-2.08) | (2.92-3.14) | (3.29-3.55) | (3.92-4.57) | (5.75-7.49) | (4.63-7.41) |

| **Table S10: Cox proportional hazard ratios (HR) with 95% confidence intervals (95% CI) for COVID 19-deaths and SARS-CoV-2 infections according to vaccination status from November 1st to December 31st, 2022, in the entire study cohort excluding nursing home residents** | | | | |
| --- | --- | --- | --- | --- |
|  | Four vaccine doses | Three vaccine doses | One or two vaccine doses | Unvaccinated |
|  | COVID-19 deaths | | | |
| COVID-19 deaths (n) | 11 | 10 | 2 | 9 |
| Events per 100.000 person days | 0.04 | 0.01 | 0.00 | 0.01 |
| Age and gender adjusted HR (95% CI) | 1.03 (0.43 - 2.45) | Reference | 0.71 (0.16 - 3.24) | 2.55 (1.03 - 6.29) |
|  | SARS-CoV-2 infections | | | |
| SARS-CoV-2 infections (n) | 7,618 | 37,131 | 22,402 | 20,217 |
| Events per 100.000 person days | 26.92 | 43.57 | 41.13 | 27.82 |
| Age and gender adjusted HR (95% CI) | 0.81 (0.79 - 0.83) | Reference | 0.94 (0.93 - 0,96) | 0.65 (0.64 - 0.67) |

| **Table S11: Cox proportional hazard ratios (HR) with 95% confidence intervals (95% CI) for all-cause deaths according to vaccination status from November 1st to December 31st, 2022, in the entire study cohort excluding nursing home residents** | | | | |
| --- | --- | --- | --- | --- |
|  | Four vaccine doses | Three vaccine doses | One or two vaccine doses | Unvaccinated |
| All cause deaths (n) | 724 | 1,214 | 467 | 463 |
| Events per 100.000 person days | 2.54 | 1.41 | 0.85 | 0.63 |
| Age and gender adjusted HR (95%) | 0.63 (0.57 - 0.69) | Reference | 1.22 (1.10 - 1.36) | 1.03 (0.93 - 1.15) |

| **Table S12: COVID-19 deaths and all-cause deaths according to vaccination status and nursing home residence status from November 1 to December 31, 2022, in the entire study cohort** | | | |
| --- | --- | --- | --- |
| **Vaccine doses** | **Nursing home residency** | **Number of COVID 19 deaths (%)** | **Number of all-cause deaths (%)** |
| **Four vaccine doses** | No | 11 (35.5) | 724 (46.2) |
|  | Yes | 20 (64.5) | 844 (53.8) |
| **Three vaccine doses** | No | 10 (50) | 1,214 (69.5) |
|  | Yes | 10 (50) | 534 (30.5) |
| **One or two vaccine doses** | No | 2 (28.6) | 467 (76.9) |
|  | Yes | 5 (71.4) | 140 (23.1) |
| **Unvaccinated** | No | 9 (81.8) | 463 (81.8) |
|  | Yes | 2 (16.2) | 103 (18.2) |
| **All** | No | 32 (46.4) | 2,868 (63.9) |
|  | Yes | 37 (53.6) | 1,621 (36.1) |

| **Table S13: Cox proportional hazard ratios (HRs) with 95% confidence intervals (95% CIs) for all-cause deaths in November 2022 and December 2022, comparing the group with four vaccinations versus groups with three, one to two and no vaccination** | | |
| --- | --- | --- |
|  | November 2022 | December 2022 |
|  | Age and gender adjusted HRs (95% CIs) for the group with four vaccinations as compared to the respective reference group | |
| Three vaccine doses (reference) | 0.80 (0.72 - 0.89) | 0.80 (0.73 - 0.87) |
| One or two vaccine doses (reference) | 0.68 (0.58 - 0.79) | 0.72 (0.63 - 0.81) |
| Unvaccinated (reference) | 0.93 (0.79 - 1.09) | 0.89 (0.78 - 1.01) |

**Figure S1: Participant selection chart**

General population of Austria as of October 1, 2022 (n= 9,090,868 )

Excluded (n=246)

♦ Inconsistent mortality data as these persons were recorded as a COVID-19 death between January 1, 2020 and October 31, 2022 but had no record as a total mortality event in the national mortality registry

Excluded (n=1)

♦ Inconsistent mortality data as this person was recorded as a COVID-19 death between November 1 and December 31, 2022 but had no recorded as a total mortality event in the national mortality registry

Study population (n=3,986,312 )

Excluded (n= 181,735 )

♦ SARS-CoV-2 infection within 90 days

before November 1, 2022

Individuals with a previous SARS-CoV-2 infection before August 3, 2022, residence in Austria, and alive on November 1, 2022 (n=4,167,294)
